# No Partner, No Children? Union Formation, Assortative Mating, and Educational Inequalities in Fertility in Germany

**DOI:** 10.1007/s10680-026-09766-w

**Published:** 2026-02-03

**Authors:** Julia Leesch, Nicole Hiekel

**Affiliations:** Max Planck Institute for Demographic Research, Independent Research Group “Gender Inequalities and Fertility”, Rostock, Germany

**Keywords:** Education, Fertility, Union formation, Assortative mating, Homogamy

## Abstract

Education is linked to whether and with whom individuals form partnerships. However, it is largely unclear how educational differences in union formation and matching with equally, more, or less educated partners shape educational disparities in fertility. Using data from the German Socio-Economic Panel, we applied marginal structural models to investigate how higher education affects the probabilities of ever having a first and a second child, and to what extent these effects operate through union formation (having a partner or not) and assortative mating (having a highly educated partner or not). We compared women and men socialised in East and West Germany before reunification, two contexts with distinct gender and stratification regimes. Findings indicate that higher education influenced fertility directly and indirectly through partnership formation, but only marginally through partners’ education. Moreover, findings varied across contexts. In West Germany, higher education reduced women’s probability of becoming mothers but had minimal impact on second births. For men, higher education increased the probabilities of having both a first and a second child. These gendered effects emerged because higher education influenced fertility directly and indirectly via union formation. In East Germany, higher education had limited effects on fertility, except for increasing men’s second birth probabilities. These weak effects resulted from small direct effects, counterbalanced by indirect effects linking higher education and fertility through union formation and assortative mating. Our study contributes to debates about precursors to family formation, underscoring the importance of union formation as a mechanism linking education and fertility.

**Supplementary Information:**

The online version contains supplementary material available at 10.1007/s10680-026-09766-w.

## Introduction

Education is one of the most important predictors of women’s and men’s fertility. Although educational differences in women’s transition to parenthood have declined or even reversed in some countries over the past decades, in most contexts a clear pattern persists (Beaujouan et al., 2016; Jalovaara et al., 2019). Compared to less educated women, highly educated women remain childless more often, have fewer children, and postpone the first birth to a higher age (Beaujouan et al., 2016; Kravdal & Rindfuss, 2008; Nisén et al., 2021; Wood et al., 2014). In contrast, for men, the pattern is the opposite; most studies indicate that low-educated men are more often childless than those who are highly educated (Jalovaara et al., 2019; Kravdal & Rindfuss, 2008; Nisén et al., 2014; Trimarchi & Van Bavel, 2017b).

Understanding the causes of these educational gradients in female and male fertility is crucial, as they can have far-reaching consequences for population dynamics and social inequalities within and between generations. Differences in fertility by educational attainment may have driven recent fertility trends, given the profound expansion of tertiary education over the past decades (Gray & Evans, 2019; Lazzari et al., 2021). Moreover, educational disparities in fertility have the potential to impact the transmission of educational inequalities across generations. Childlessness, for example, hinders individuals from transmitting their socioeconomic resources to the next generation (Lawrence & Breen, 2016; Skopek & Leopold, 2020; Wittemann & Yastrebov, 2024). However, parents with larger families may also struggle to pass on their socioeconomic position due to resource dilution, meaning they must distribute their resources among multiple children (Blake, 1989; Kalmijn & Van De Werfhorst, 2016).

Although educational disparities in fertility can have considerable implications for demographic change and social stratification, the drivers of these disparities remain insufficiently understood. Fertility is routinely studied through an individualistic lens, with much of the research focusing on whether individuals can afford children, their opportunity costs, or personal values and attitudes toward family formation (Beaujouan et al., 2016; Becker, 1981; Lesthaeghe, 2010; Mincer, 1963; Wood et al., 2014). However, scholars have often overlooked that education sorts individuals into social contexts, such as partnerships, that influence their probability of having children.

Education may affect fertility not only directly, but also indirectly by influencing *whether* someone has a partner, and *who* the partner is. First, education could shape fertility through selection into partnerships. In many contexts, studies documented a positive educational gradient in men’s union formation and marriage, and a negative gradient for women (Bertrand et al., 2020; Cantalini, 2017; Goldstein & Kenney, 2001; Kalmijn, 2013), which however reversed in many countries among younger women (Bertrand et al., 2020; Sturm & Van Bavel, 2024). These union formation patterns can reduce fertility among the educational groups that are more often unpartnered. Second, education could affect fertility through educational assortative mating, the tendency to form unions with similarly educated partners (Kalmijn, 1998; Schwartz, 2013; Schwartz & Mare, 2005). As higher education increases the probability of having a highly educated partner, it also shapes the couple’s joint resources, opportunity costs, and values, which may mediate the relationships between women’s and men’s education and their fertility outcomes. To address these mechanisms, we investigated two research questions: “*How does higher education affect women’s and men’s lifetime probabilities of having a first and a second child?”* and *“To what extent do union formation and educational assortative mating mediate these effects?”*.

Previous research increasingly recognised that union formation is a pathway through which education may affect fertility (Jalovaara et al., 2019; Trimarchi & Van Bavel, 2017a). However, empirical research is sparse. While Trimarchi and Van Bavel (2017a) showed that less educated men were less likely to become fathers because they were less likely to have a partner; little is known about whether the same applies to women. Furthermore, it is largely unclear whether the effects of education on women’s and men’s fertility also operate through assortative mating. These are notable gaps, especially for comparative fertility research, because cross-country differences in the link between education and fertility may arise from differences in union formation and assortative mating.

Drawing on data from the German Socio-Economic Panel (SOEP), we analysed 2,618 women and 2,479 men born between 1959 and 1975 in East and West Germany. The historical division between East and West Germany from 1949 to 1990 offers a unique setting for comparative fertility research. While East Germany’s state-socialist regime actively promoted gender equality in education and labour force participation, West German policies incentivised the male breadwinner model, increasing women’s opportunity costs of childbearing. For the empirical analysis, we employed marginal structural models, a method widely used in epidemiology but novel to fertility research. This approach allows us to address an important gap in fertility research by analysing to what extent the effect of education on lifetime fertility is mediated by having no partner, a highly educated partner, or a less educated partner.

## Background

Research on educational differences in fertility has largely been guided by theoretical frameworks that suggest education *directly* influences fertility through economic and ideational mechanisms.^1^ Three core perspectives dominate the literature. First, standard economic theory, assuming a strictly gendered division of labour, predicts gender-specific effects of education on fertility through two opposing mechanisms (Becker, 1981). The income effect suggests that higher education enhances fertility by increasing financial resources for parenting, while the opportunity cost effect implies that education reduces fertility by raising the costs of time spent on unpaid caregiving rather than paid work. These mechanisms predict a negative educational gradient in fertility for women and a positive one for men.

Second, another line of research emphasises the role of economic uncertainty and labour market constraints for family formation (Blossfeld et al., 2006; Oppenheimer, 1988). Globalisation and technological advancements are thought to have contributed to the rise of precarious employment and earnings declines, especially among the less educated (De Vries et al., 2020; Kalleberg, 2009, 2011). As a result, low-educated women and men increasingly struggle to signal their future socioeconomic status on the partner market and to commit financially to the resource-intensive phase of parenthood (Oppenheimer, 1988; Schwartz, 2013).

Third, the Second Demographic Transition (SDT) framework argues that ideational change, particularly the rise in values associated with individual autonomy, self-realisation, and gender egalitarianism, led to prolonged periods of singlehood and cohabitation, later fertility, and greater acceptance of childlessness (Lesthaeghe, 2010). The SDT hypothesises that this value change was more pronounced among the highly educated, leading to delayed union formation and fertility, as well as greater childlessness among better-educated women and men.

Although these theories suggest that education influences union formation *and* fertility, they pay limited attention to union formation and assortative mating as channels through which education may *indirectly* affect fertility. Yet economic, uncertainty-based, and ideational theories all imply that education could influence fertility not only through preferences and constraints, but also through differential access to partners. For example, if rising economic uncertainties reduced union formation rates among the low-educated, we can expect that this would lower fertility levels in this group.

Generally, education is associated with whether and with whom individuals form partnerships, which may either facilitate or constrain fertility. Yet, empirical research rarely examined how education influences fertility through union formation and assortative mating. This limits not only our general understanding of how education affects fertility, but it is also influential for comparative fertility research. Although many contexts show a negative educational gradient in women’s fertility (Beaujouan et al., 2016; Kravdal & Rindfuss, 2008; Wood et al., 2014) and a positive educational gradient in men’s fertility (Jalovaara et al., 2019; Trimarchi & Van Bavel, 2017a), the education-fertility association differs across contexts (Vasireddy et al., 2023). For example, educational inequalities in fertility are less pronounced in East than in West Germany (Kreyenfeld, 2004; Sobotka, 2012). Union formation and assortative mating may help explain such contextual variation because the educational gradient in union formation and patterns of educational assortative mating vary substantially across institutional contexts (Domański & Przybysz, 2007; Kalmijn, 2013; Smits, 2003; Sturm & Van Bavel, 2024).

We distinguish between two pathways that may mediate the effect of education on fertility. First, education could influence fertility indirectly through selection into unions. Until recently, highly educated women and low-educated men were typically overrepresented among the unpartnered (Bertrand et al., 2020; Cantalini, 2017; Goldstein & Kenney, 2001; Kalmijn, 2013). Yet, in younger cohorts, the negative educational gradient in women’s union formation often reversed (Bertrand et al., 2020; Sturm & Van Bavel, 2024). Consequently, the positive educational gradient in men’s union formation and the historically negative gradient in women’s union formation may help explain the respective educational gradients in fertility.

Although many studies examined the link between education and union formation, few directly investigated whether union formation mediates the effect of education on fertility. A notable exception is the study by Trimarchi and Van Bavel (2017a); they found a positive effect of education on the transition to fatherhood that operates through selection into unions. However, other studies, which primarily analysed how education reproduces across generations – with union formation, assortative mating, and fertility as potential mediators – report mixed findings. Results range from union formation having negligible effects to it being the primary mechanism linking education and first births (Corti & Scherer, 2022; Lawrence & Breen, 2016). These inconsistencies underline the need for further research on union formation as a mediating path between education and fertility.

Second, education could affect fertility indirectly through assortative mating, as different educational pairings face varying economic and normative constraints. Several studies have examined how both partners’ education relates to fertility transitions (Bueno & García-Román, 2021; Corijn et al., 1996; Dribe & Stanfors, 2010; Jalovaara & Miettinen, 2013; Nitsche, 2024; Nitsche et al., 2018). Taken together these studies find that unions between two highly educated partners delay first births longer than other pairings, but are more likely to proceed to higher-order births. However, findings vary across contexts. For example, in some Central and Eastern European countries, such unions were not associated with higher second birth rates (Trimarchi & Van Bavel, 2020).

While these dyadic studies offer valuable insights by showing that fertility patterns vary across educational pairings, they typically condition on union formation, which is itself a potential mediator of the effect of education on fertility (see Trimarchi & Van Bavel, 2017a). As a result, they do not capture the total effect of education on fertility among partnered and unpartnered women and men. Furthermore, controlling for both partners’ education can block a causal pathway through which education affects fertility. Because partners often have similar educational levels, higher education could influence fertility by increasing the chances of forming unions with highly educated partners (see Grätz, 2022).

Another line of research applied marginal structural models, which help address methodological challenges related to conditioning on mediators. While these studies primarily investigated the intergenerational reproduction of education, they also provide insights relevant to our question. In an intermediary step, they show that the partner’s education contributed only moderately to the total effect of higher education on the probability of having the first child (Corti & Scherer, 2022; Lawrence & Breen, 2016). However, this evidence offers limited insight into how education influences higher-order births via assortative mating and whether these findings vary across institutional contexts.

To conclude, education may influence fertility directly through income, opportunity costs, or value orientations, and indirectly, by shaping who enters a union and with whom. Building on previous research, we expect higher education to reduce fertility for women and increase it for men (hypothesis 1). In our temporal and geographic context, we further expect highly educated women and less educated men to be less likely to have children because they are less likely to have a partner (hypothesis 2). At the same time, assortative mating may counterbalance these effects because individuals whose education is linked to higher fertility often partner with those whose education is linked to lower fertility (e.g., highly educated men tend to form unions with highly educated women) (hypothesis 3).

## The German Context

The role of education in shaping fertility varies across social and institutional settings. Germany offers a unique case to examine how higher education influenced union formation and fertility among individuals raised in the contrasting socio-political systems of the German Democratic Republic (GDR) and the Federal Republic of Germany (FRG). Despite a shared language and cultural heritage, the two regimes developed distinct educational policies, welfare systems, and family and gender norms (Kreyenfeld, 2004). Our study focused on individuals aged 14 to 30 at reunification, ensuring they were exposed to policies and norms that had evolved over four decades in separate regimes. Thus, although East Germany adopted West Germany’s legal and political systems after reunification, our temporal framework allows us to explore how education affected fertility across these different contexts.

In West Germany, the highly stratified educational system, which tracks students into different secondary school types, is believed to perpetuate educational inequalities (Betthäuser, 2019; Bol & Van De Werfhorst, 2013), making education a crucial indicator for career prospects, earnings, and the affordability of children. Moreover, the FRG represented an ideal-type conservative welfare state, with family policies reinforcing a male-breadwinner model. Parental leave policies provided minimal wage replacement, childcare options were limited, particularly for children under three, part-time care in schools hindered mothers’ labour market integration, and tax policies further incentivised unequal spousal earnings (Kreyenfeld, 2004). Consequently, these measures likely contributed to a positive educational gradient in men’s fertility and a negative one in women’s, because motherhood resulted in high opportunity costs for highly educated women.

In contrast, the GDR promoted egalitarian educational and family policies. All children attended the same schools typically until grade ten, and quotas facilitated access to higher education for children from working-class backgrounds (Betthäuser, 2019; Huinink, 1995; Von Below, 2002). The state constitutionally guaranteed the right to work and supported women’s fertility and labour force participation, through full-time childcare, generous parental leave, and marriage loans partially forgiven upon childbearing (Kreyenfeld, 2004; Trappe, 1995). In addition, housing policies prioritised families by offering subsidised rents and giving preference to married couples and parents in housing allocations (Schulz, 2010; Winkler, 1990). By promoting mothers’ full-time employment, these measures fostered gender equality in the relationship between education, union formation, and fertility. Additionally, broad access to jobs, childcare, and housing may have reduced the impact of education on economic well-being and the ability to afford a family. Consequently, the GDR’s policies may have led to less pronounced education effects on union formation and fertility.

Empirical evidence shows pronounced educational disparities in women’s fertility in West Germany: highly educated women had fewer children, higher childlessness, and delayed first births. In East Germany, such differences were weaker or absent (Kreyenfeld, 2004; Kreyenfeld & Konietzka, 2017; Skopek & Leopold, 2020; Sobotka, 2012). Less is known about men’s educational gradients in fertility in East and West Germany. However, available findings suggest only minor differences in the average number of children between men with and without upper secondary degrees in both regions (Skopek & Leopold, 2020).

Research on East-West differences in educational inequalities in union formation and assortative mating is limited. Wirth (2000), for example, found a negative educational gradient in marriage for West German women, but only moderate differences in East Germany. Yet, studies examining educational gradients in union formation across all co-residential unions are lacking, which is a considerable gap, given that cohabitation is more often an alternative to marriage in East Germany (Hiekel et al., 2015). Furthermore, evidence on East-West differences in relative homogamy, net of the educational composition, is inconclusive. However, the proportion of educationally homogamous unions is higher in East Germany, possibly due to less variation in educational attainment (Grave & Schmidt, 2012; Konietzka & Kreyenfeld, 2002; Wirth, 2000).

Taken together, research on East-West differences in the relationship between education, union formation, assortative mating, and fertility is sparse; robust findings exist only for women’s fertility. Nonetheless, given the differences in institutional contexts, we hypothesise that education generally more strongly stratifies union formation, assortative mating, and fertility in West than in East Germany (hypothesis 4).

## Data and Methods

### Data and Sample

We used data from the German Socio-Economic Panel (SOEP), including main and refresher samples from East and West Germany (Goebel et al., 2019, 2023). The SOEP is one of the longest-running panel studies worldwide, with annual interviews conducted since 1984 in West Germany and since 1990 in East Germany (Goebel et al., 2019).

Our analyses focused on women and men born between 1959 and 1975. These birth cohorts are well-suited for our study because they allow us to observe completed fertility: the youngest respondents, born in 1975, had reached age 45 by 2020. Moreover, the oldest respondents, born in 1959, were young enough to capture most of their adult partnership histories.

We constructed a dataset consisting of a single observation per respondent, capturing their education and the number of children they had after reaching age 45. The respondents in our sample reached age 45 between 2004 and 2020. If the observation at age 45 was missing, we used the next available one between the ages of 46 and 50. Moreover, the dataset captures whether respondents had any partner, and if applicable, the educational attainment of the most recent partner. Information on the partner’s education may stem from earlier waves, as respondents’ most recent partnership may have ended before they reached age 45.

The initial sample included 5,888 individuals. From this sample, we excluded respondents with missing partner data^2^ (*n* = 497), unknown or foreign residence in 1989 (i.e., the fall of the Berlin Wall), (*n* = 308), or missing education data (*n* = 4). The final analytical sample comprises 5,097 women and men.

### Measurement

#### Fertility

We used two dichotomous measures of fertility outcomes. The first measure indicates whether individuals had biological children, while the second differentiates between parents with one child and those with two or more biological children. We chose these measures because we were not only interested in the transition to parenthood, but also whether partner search outcomes explain educational gradients in achieving approximately replacement fertility and thus contributing to the stabilisation or decline of the population size.

#### Education

Higher education, the treatment variable, was measured dichotomously: tertiary degree (ISCED 5 to 6) versus no tertiary degree (ISCED 1 to 4). We measured educational attainment at the end of the reproductive phase, but for most respondents (92.8%), their educational level remained constant throughout their reproductive period. For those with educational upgrades, we used the most recent educational level, as couples often anticipate educational advancements when making fertility decisions. When educational attainment was missing at the last observation, we relied on information from earlier years.

#### Partner Search Outcomes

To measure partner search outcomes, we classified each individual into one of three categories based on their union status and the educational attainment of their most recent partner: (1) never partnered, (2) low-educated partner, or (3) highly educated partner. Because most children are born within co-residential unions, we applied a narrow definition of partnerships, including only cohabitation and marriage. To identify such unions, we used partner identifiers that link respondents to co-resident partners. Individuals who did not report any co-residential union were classified as unpartnered. For those who were ever partnered, we used the educational attainment (tertiary versus non-tertiary) of the most recent partner. To identify the most recent partner, we traced partner histories back to age 25.

Because our analytical approach swaps the probabilities of experiencing these partner search outcomes between educational groups, we cannot portray complex partnership trajectories that may emerge through union dissolution and repartnering. Consequently, our measure, which is based on the education of the most recent partner, may overlook earlier exposure to partners with different educational levels. That means, in some cases, individuals may have had children with an earlier partner whose educational level differed from that of their most recent partner. However, since assortative mating also occurs in repartnering, and we categorised education into only two groups, we assume that even if someone’s most recent partner was not their child’s parent, they often had the same educational level (see Breen & Ermisch, 2017). Empirically, this assumption holds; in our sample, fewer than 7 per cent of individuals experienced changes in their partner’s education, either due to repartnering or because their partner attained a higher degree later in life.

#### Covariates

We measured parents’ socioeconomic status (SES), as parental SES predicts children’s likelihood of attaining a higher degree (e.g., Breen, 2004; Erikson & Goldthorpe, 1992). Our SES measure is based on the Standard International Socio-Economic Index (ISEI), selecting the higher SES parent when data for both parents were available and, alternatively, the SES of the available parent. Second, as sibship size may affect children’s educational attainment (e.g., through resource dilution), we accounted for the number of siblings, categorised as 0, 1, 2, and 3 or more. Third, we considered regional differences, distinguishing between individuals who grew up in urban and rural areas (classified as large city, medium-sized city, small town, or rural area). And lastly, we accounted for migration background, distinguishing between no migration background (respondent and both parents born in Germany), direct migration background (respondent born abroad), and indirect migration background (respondent born in Germany and at least one parent born abroad).

### Methods

To examine the effect of tertiary education on the probabilities of having a first and a second child, we employed marginal structural models with inverse probability of treatment weighting (IPTW) (Hernán & Robins, 2023; Robins, 1999; VanderWeele, 2009). Unlike traditional mediation analyses, which estimate controlled direct effects by holding the mediator constant at a specific value, marginal structural models allow us to study theoretically more meaningful counterfactuals by estimating *natural* direct and indirect effects. As we illustrate below, marginal structural models analyse the marginal distribution of counterfactual variables (Robins, 1999). This approach enables us to estimate natural direct effects, which refer to the effect of the treatment on potential outcomes, assuming the mediator remains at the level that naturally occurs without treatment. For instance, we may ask: How would the treatment (tertiary education) affect the probability of having a first child if the treated group had the same probability of being unpartnered as the untreated group? In contrast, natural indirect effects reflect how the outcome changes when the mediator shifts from the level it has without treatment to the level it takes when the treatment is in place (Pearl, 2022).

Following Lawrence and Breen (2016), our analysis involved three steps, which we executed separately for women and men from East and West Germany. First, we adjusted for selection into treatment. We used IPTW to create a pseudo population in which higher education is no longer confounded by observed covariates. To do so, we estimated propensity scores by regressing tertiary education on the observed covariates using logistic regressions. Table A1 in the Online Appendix shows these regression results. Subsequently, we calculated IPTWs from these propensity scores.^3^ We applied a similar approach to account for selection into different types of unions and first births. We provide a detailed description of the construction of all weights in the Appendix. Moreover, Table A2 in the Online Appendix shows the distribution of all IPTWs^4^. Missing data on categorical variables (e.g., siblings) were handled by including a separate category for missing values. For continuous variables (e.g., parents’ SES), missing values were set to 0, with an indicator variable to flag missing cases (Choi et al., 2019). Missing data on covariates ranges from approximately 10% for parents’ SES to complete information for migration background.

Second, we applied linear probability models to this weighted data to estimate four marginal structural models (Eqs. 1, 2, 3, 4) and obtain all direct effects required for our analysis. In Eq. 1, $\:{\alpha\:}_{1}$ captures the direct effect of higher education (A) on the potential outcome of having no partner (Z0). Equation 2 models the probability of having a highly educated partner (Z2), with $\:{\gamma\:}_{1}\:$representing the direct effect of higher education (A). In Eq. 3, $\:{\beta\:}_{1\:}$estimates the direct effect of higher education (A) on the probability of having the first child (C), while $\:{\beta\:}_{2}$ and $\:{\beta\:}_{3}$ correspond to the direct effects of having no partner (Z0) and having a highly educated partner (Z2), respectively. Having a partner without a tertiary degree (Z1) serves as the reference category. Similarly, in Eq. 4, we estimated the direct effects of higher education (A), no partner (Z0), and a highly educated partner (Z2) on the probability of having a second child (Y). For estimating Eq. 4, we used data solely from individuals who already had a child, yet due to the counterfactual design, it also indicates the expected or counterfactual effect for childless individuals.

1 $$E\left( {Z{O^A}} \right)={\alpha _0}+{\alpha _1}A$$

2 $$E\left( {Z{2^A}} \right)={\gamma _0}+{\gamma _1}A$$

3 $$E\left( {{C^{A,Z0,Z2}}} \right)={\beta _0}+{\beta _1}A+{\beta _2}{Z_0}+{\beta _3}{Z_2}$$

4 $$E\left( {{Y^{A,Z0,Z2}}} \right)={\theta _0}+{\theta _1}A+{\theta _2}{Z_0}+{\theta _3}{Z_2}$$

Third, we used the coefficients from these models (Eqs. 1, 2, 3, 4) to compute the indirect effects of tertiary education on fertility. Equation 5 estimates how higher education affects the probability of having a first child through union formation and assortative mating. For capturing this indirect effect, we compared two potential outcomes: in the first term of Eq. 5, we set the treatment to zero (A = 0) and both partner search outcomes (no partner and highly educated partner) to the level that occurs when the treatment is in place (A = 1); in the second term the treatment and mediators were set to zero (A = 0).

This indirect effect can be decomposed into two components: the effect mediated by union formation (Eq. 6) and the effect mediated by having a tertiary-educated partner (Eq. 7). This decomposition is an extension of previous research, which examined the indirect effect as a compound measure, as shown in Eq. 5 (Corti & Scherer, 2022; Lawrence & Breen, 2016). Similarly, we calculated the indirect effect of education on the progression from the first to the second child by replacing *C* with *Y* and $\:\beta\:\:$with $\:\theta\:$.567

**Fig. 1:**
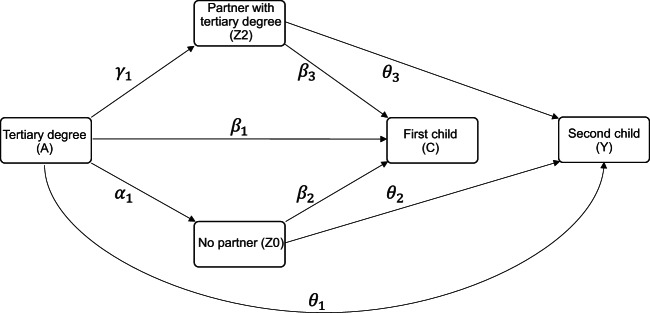
Total, direct, and indirect effects of higher education on **a** having the first child, and **b** progressing to the second child

Figure 1 provides a graphical illustration of the models presented in Eqs. 1, 2, 3, 4, 5, 6, 7. Each arrow represents a direct effect. For example, $\:{\gamma\:}_{1}$ indicates the effect of higher education on the probability of having a tertiary-educated partner. All estimates for indirect effects are based on a combination of treatment effects on the mediator and mediator effects on the outcome (Eqs. 5, 6, 7). For example, suppose that tertiary education reduces the probability of being unpartnered by 10% points (= −0.10) and being unpartnered reduces the probability of having the first child by 80% points (= −0.80), then the indirect effect through union formation would be 8% points (−0.10 ⋅ −0.80 = 0.08). That means, highly educated individuals would have a higher probability of having the first child, partly because they are less likely to be unpartnered.

Lastly, we estimated the realised effects of higher education on having two or more children. Since having a second child is only possible after the first birth, two causal effects shape this realised effect: the effects of higher education on (a) having a first child, and (b) progressing from a first to a second child. The realised effect combines them by multiplying the probabilities of both transitions (Lawrence & Breen, 2016). For example, the *total* realised effect of higher education on having at least two children is calculated in Eq. 8. All standard errors were obtained via nonparametric bootstrapping with 500 replications.

8 $$ \begin{gathered} E\left( {Y^{{A = 1,Z0\left( {A = 1} \right),Z2\left( {A = 1} \right)}} } \right)E\left( {C^{{A = 1,Z0\left( {A = 1} \right),Z2\left( {A = 1} \right)}} } \right) \hfill \\ \quad - E\left( {Y^{{A = 0,Z0\left( {A = 0} \right),Z2\left( {A = 0} \right)}} } \right)E\left( {C^{{A = 0,Z0\left( {A = 0} \right),Z2\left( {A = 0} \right)}} } \right) \hfill \\ \end{gathered} $$

### Threats to Causality

In this section, we address the assumptions underlying our causal estimates. Our first assumption is the absence of reverse causality, where fertility influences education. For instance, individuals who have children early may forgo pursuing higher education. Fertility might also affect a partner’s education, in cases where unintended pregnancies lead to union formation with the child’s father. While these scenarios are plausible, only a small proportion of births occur to parents who married during the pregnancy (Gibson-Davis et al., 2016; Rackin & Gibson-Davis, 2012). Moreover, multiple studies indicate that educational expansion led to a postponement of fertility (e.g., Liefbroer & Corijn, 1999; Ní Bhrolcháin & Beaujouan, 2012), suggesting that education affects fertility more than the other way around.

The second assumption is the absence of omitted variable bias. While unobserved factors, such as personal preferences or broader social norms, may simultaneously influence education, partner search, and fertility decisions, our inclusion of extensive controls, such as parents’ SES, number of siblings, and migration background, helps mitigate this risk as far as possible given the available data.

We also recognise the potential role of unmeasured mediators, such as union dissolution, in shaping fertility outcomes. For example, educational differences in union dissolution could partially explain the effect of the partner’s education on fertility. That means, while we might find that the partner’s education affects fertility, we did not investigate the underlying reasons, such as union dissolution, linking the partner’s education and fertility outcomes.

Despite these challenges, our approach improves upon prior research. Many existing studies are limited by selection bias, often analysing only partnered individuals, which can distort conclusions about the full population. Additionally, previous research frequently classified partners’ education as a confounder rather than a mediator, potentially leading to overcontrol bias (e.g., Corijn et al., 1996; Jalovaara & Miettinen, 2013; Nitsche et al., 2018, 2021). By accounting for selection processes that occur during the partner search stage and precede family formation, we offer a more inclusive analysis that advances the field.

## Results

### Descriptive Results

Table 1 provides an overview of union formation and fertility outcomes by gender, region, and education. In West Germany, higher education was negatively associated with fertility outcomes for women, and positively for men. For example, 27.1% of highly educated West German women remained childless, compared to only 16.8% of less educated women. Moreover, tertiary-educated women and non-tertiary-educated men were overrepresented among the unpartnered. This suggests that union formation could be a mechanism linking education and fertility in West Germany. Additionally, highly educated women and men were more likely to have highly educated partners, indicating that educational assortative mating may influence how higher education affects fertility.

**Table 1:** Union formation and fertility outcomes by gender and education

		Partner				First child			Second child		
		Partner no degree	Partner with degree	No partner	Total	No	Yes	Total	No	Yes	Total
Women West Germany	No tertiary degree	888 65.88	389 28.86	71 5.27	1348 100.00	226 16.77	1122 83.23	1348 100.00	510 37.83	838 62.17	1348 100.00
	Tertiary Degree	142 27.68	316 61.60	55 10.72	513 100.00	139 27.10	374 72.90	513 100.00	241 46.98	272 53.02	513 100.00
Men West Germany	No tertiary degree	794 74.69	145 13.64	124 11.67	1063 100.00	286 26.90	777 73.10	1063 100.00	513 48.26	550 51.74	1063 100.00
	Tertiary Degree	382 54.11	290 41.08	34 4.82	706 100.00	143 20.25	563 79.75	706 100.00	261 36.97	445 63.03	706 100.00
Women East Germany	No tertiary degree	342 70.37	110 22.63	34 7.00	486 100.00	45 9.26	441 90.74	486 100.00	202 41.56	284 58.44	486 100.00
	Tertiary Degree	125 46.13	131 48.34	15 5.54	271 100.00	26 9.59	245 90.41	271 100.00	110 40.59	161 59.41	271 100.00
Men East Germany	No tertiary degree	330 66.67	117 23.64	48 9.70	495 100.00	90 18.18	405 81.82	495 100.00	238 48.08	257 51.92	495 100.00
	Tertiary Degree	90 41.86	110 51.16	15 6.98	215 100.00	38 17.67	177 82.33	215 100.00	92 42.79	123 57.21	215 100.00

Unweighted sample distributions for respondents aged 45+. In the unweighted data, East Germans and adults without German nationality were oversampled, and no adjustments for nonresponse or calibrations to population benchmarks were applied. These figures provide descriptive context for the marginal structural models. Following prior research, unweighted results are reported to describe the analytical sample (Sharkey & Elwert, 2011; Wodtke et al., 2016).

In contrast, East Germany shows more uniform fertility outcomes across educational groups, with only one notable exception: tertiary-educated men had a higher probability of having a second child. Furthermore, highly educated women and men were slightly underrepresented among the unpartnered, suggesting that higher education may increase fertility through union formation. Moreover, similar to West Germany, we expect that assortative mating may shape the effect of higher education on fertility because higher education was associated with a greater probability of having a highly educated partner. Overall, these regional disparities indicate that the interplay between education, union formation, and fertility may differ between East and West Germany.

### Marginal Structural Models

**Fig. 2:**
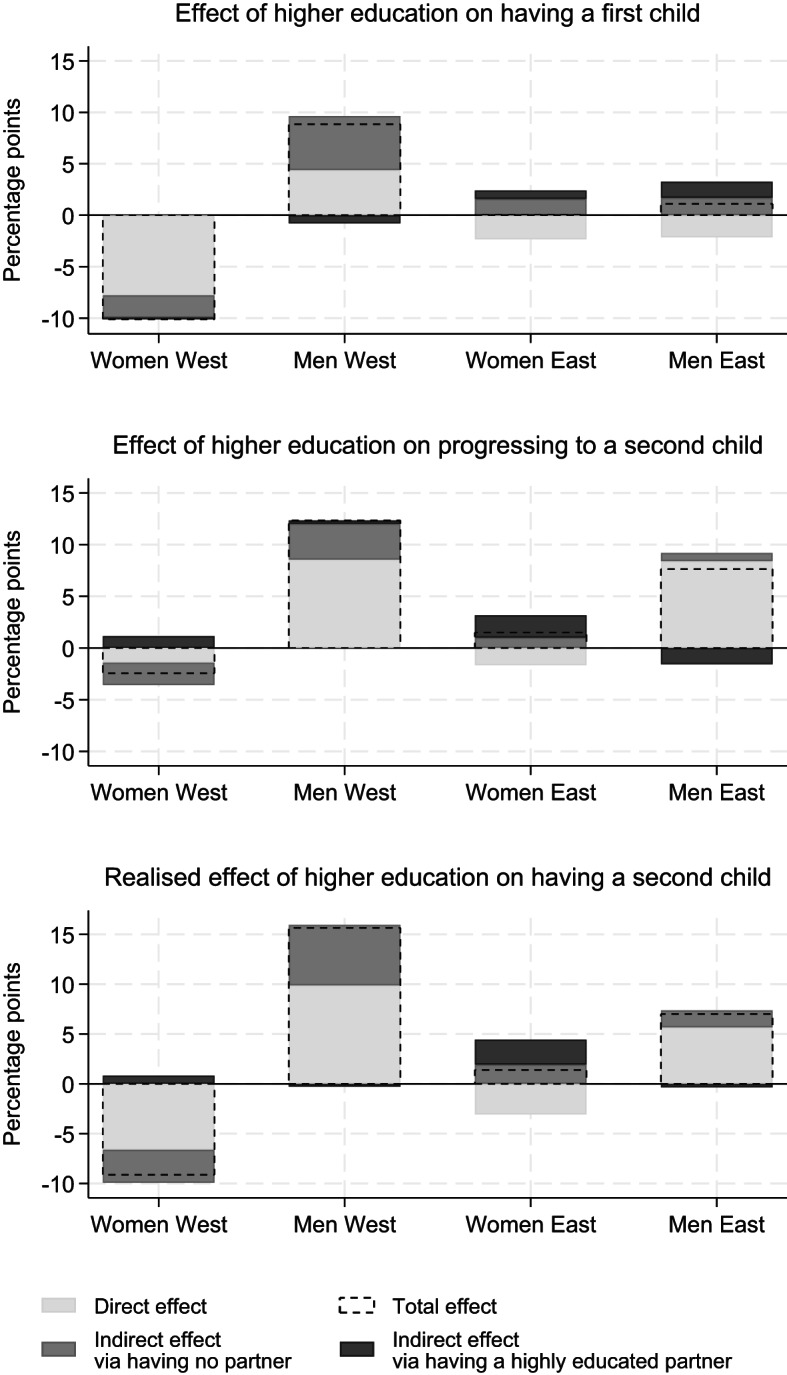
Total, direct, and indirect effects of higher education on having a first child (C), progressing to a second child (Y), and having a second child (Realised Y). Note: Direct and indirect effects add up to the total effects. Outcomes: having a first child (probability of having a first child), progressing to a second child (probability of progressing from a first to a second child), having a second child (product of the probability of having a first child and the probability of progressing to a second child)

Figure 2 shows the total, direct, and indirect effects of higher education on fertility outcomes separately for women and men from East and West Germany. Exact estimates and standard errors are presented in Table A3 in the Online Appendix.

For *West German women*, higher education reduced the probability of becoming a mother by 10.1% points compared to less educated women. Despite the large magnitude of this effect, it is not statistically significant. The indirect effect, mediated through union formation and assortative mating, accounted for 2.4% points of this effect, primarily driven by selection into unions (−0.022), while the effect through partnering with a highly educated man was negligible (−0.002).

Table 2 provides the parameter estimates underlying these indirect effects. Highly educated West German women were considerably more likely to have highly educated partners (0.281), but this had a minimal impact on their probability of becoming mothers (0.281 ⋅ −0.006 = −0.002). In contrast, higher education increased the probability of being unpartnered only moderately (0.040), but this produced an indirect effect of −2.1% points (0.040 ⋅ −0.532 = − 0.021) because being unpartnered was a strong predictor of childlessness (−0.532).

**Table 2:** Parameter estimates from marginal structural models

	Women, West Germany				Men, West Germany			
	(1) Z0	(2) Z2	(3) C	(4) Y	(1) Z0	(2) Z2	(3) C	(4) Y
A	0.040^**^ (0.015)	0.281^***^ (0.029)	−0.078^**^ (0.025)	−0.014 (0.029)	−0.069^***^ (0.013)	0.244^***^ (0.023)	0.043^*^ (0.020)	0.086^**^ (0.027)
Z0			−0.532^***^ (0.047)	−0.535^***^ (0.070)			−0.767^***^ (0.022)	−0.510^***^ (0.121)
Z2			−0.006 (0.021)	0.041 (0.026)			−0.033 (0.025)	0.013 (0.030)
Intercept	0.054^***^ (0.006)	0.307^***^ (0.013)	0.857^***^ (0.013)	0.744^***^ (0.017)	0.117^***^ (0.010)	0.143^***^ (0.012)	0.815^***^ (0.014)	0.700^***^ (0.019)
*N*	1861	1861	1861	1496	1768	1768	1768	1340

	Women, East Germany				Men, East Germany			
	(1) Z0	(2) Z2	(3) C	(4) Y	(1) Z0	(2) Z2	(3) C	(4) Y
A	−0.028 (0.018)	0.239^***^ (0.039)	−0.023 (0.022)	−0.017 (0.042)	−0.026 (0.025)	0.246^***^ (0.040)	−0.022 (0.031)	0.084 (0.048)
Z0			−0.563^***^ (0.078)	−0.358^**^ (0.124)			−0.660^***^ (0.061)	−0.307 (0.169)
Z2			0.034 (0.020)	0.090^*^ (0.043)			0.063^*^ (0.029)	−0.064 (0.045)
Intercept	0.076^***^ (0.013)	0.224^***^ (0.020)	0.939^***^ (0.014)	0.644^***^ (0.027)	0.100^***^ (0.014)	0.240^***^ (0.020)	0.869^***^ (0.019)	0.661^***^ (0.029)
*N*	757	757	757	664	710	710	710	582

A: tertiary degree, Z0: no partner, Z2: tertiary educated partner, C: first child, Y: second child. Standard errors in parentheses. Significance: *p <.05, **p <.01, ***p <.001

Higher education also slightly reduced West German women’s likelihood of having a second child, with a total effect of −2.4% points. This effect was largely explained by differences in union formation, the only statistically significant path. When combining transitions to first and second births, the realised effect of higher education on having two or more children was substantial: For West German women, higher education reduced the probability of having at least two children by 9.1% points, reflecting direct and indirect pathways. If highly educated women had the partner search outcomes of less educated women, their probability of having two or more children would have been only 6.6% points lower. In particular, union formation played a critical role, accounting for 3.3% points of the total effect.

For *West German men*, higher education had a pronounced positive effect on fertility outcomes. Tertiary education increased the chances of becoming a father by 8.8% points, with roughly equal contributions from the direct effect of higher education and the indirect effect through partner search outcomes. The indirect effect was driven by differences in union formation (−0.069 ⋅ −0.767 = 0.0529). Partnering with highly educated women counterbalanced this effect minimally.

Higher education also considerably increased the probability of West German men having a second child, by 12.4% points. The total effect of higher education was largely driven by the direct effect (8.6% points), with additional contribution from the indirect pathway through selection into unions (3.5% points). Moreover, the total realised effect of tertiary education on having at least two children was substantial: 15.7% points, with the direct effect as the dominant driver. However, the indirect effect via union formation also contributed to this effect. If less educated men had the same probability of having a partner as highly educated men, their probability of having at least two children would increase by 6.1% points.

In East Germany, the effects of higher education on fertility were less pronounced. For *East German women*, higher education had a slightly negative direct effect on having a first child, but this was counterbalanced by partner search outcomes. Highly educated women had a lower probability of remaining unpartnered, a key predictor of childlessness, and a higher probability of partnering with highly educated men, which marginally increased their chances of parenthood. A similar pattern emerged for second births: the direct effect is negative, partially offset by positive indirect effects through union formation and assortative mating.

For *East German men*, patterns in the effects of higher education on first births resembled those of women. However, for second births, higher education had a strong positive effect (0.076) compared to women (0.015). This effect was primarily driven by the direct effect of higher education, with a smaller contribution from union formation. The realised effect of tertiary education on having a second child mirrored these findings.

In sum, the results show that education affected fertility outcomes directly and indirectly through union formation. However, the direction and magnitude of these effects varied by gender, parity, and regional context. Consistent with our expectations, the findings for West Germany largely align with our predictions regarding gendered educational gradients. Higher education was negatively associated with fertility outcomes for women and positively for men, especially for the transition to the first child. Union formation mediated part of these associations. Taken together, these results support hypotheses 1 and 2 in the West German context. In contrast, we found little evidence indicating that assortative mating counterbalanced direct educational effects on fertility in West Germany, offering no support for hypothesis 3 in this context.

In East Germany, the patterns differed markedly. Higher education showed limited associations with fertility outcomes for women, while for men, only the transition to a second child was positively associated with education. Accordingly, hypotheses 1 and 2 received little support in the East German context. However, assortative mating slightly counterbalanced the direct effects of education on fertility, providing partial support for hypothesis 3. Finally, consistent with hypothesis 4, education was a stronger predictor of union formation and fertility in West than in East Germany, underscoring the importance of institutional contexts in shaping variation in educational gradients in fertility and the role of indirect pathways in explaining these differences.

## Conclusion

This study examined to what extent union formation and educational assortative mating mediated the effect of higher education on women’s and men’s lifetime probabilities of having a first and a second child. Drawing on data from individuals socialised in the distinct socio-political contexts of East and West Germany, we applied marginal structural models to estimate how the effect of higher education on fertility would change if one educational group had the partner search outcomes of another.

Our findings show that, for West Germany, higher education reduced women’s probability of having a first child but had little effect on second births. In contrast, these effects were positive for men. The effects were not explained by educational assortative mating, but partly by union formation. That means lower rates of union formation among highly educated women and less educated men partly explained why higher education was linked to lower fertility for women and higher fertility for men. In East Germany, higher education had a small, statistically insignificant effect on first-birth probabilities for both genders and second-birth probabilities for women. These limited effects reflect a combination of small direct effects and countervailing indirect effects through union formation and assortative mating. Only for men’s transition to the second child, a notable positive effect of higher education emerged, mainly driven by the direct effect.

Our study adds to existing research, reinforcing the notion that union formation plays an important role in shaping fertility (Jalovaara & Fasang, 2017; Raab & Struffolino, 2020) and serves as a mechanism linking education to fertility (Trimarchi & Van Bavel, 2017a). Moreover, our finding that assortative mating did not substantially mediate the effect of higher education on first and second-birth probabilities aligns with prior studies, showing similar findings for first births (Corti & Scherer, 2022, p. 6; Lawrence & Breen, 2016, p. 564). However, it contrasts with studies reporting higher-order fertility advantages among highly-educated homogamous couples (Dribe & Stanfors, 2010; Nitsche et al., 2018). These discrepancies may stem from conceptual and methodological differences. First, most prior work used event history models that conflate timing and quantum, whereas our approach isolates quantum fertility (Corijn et al., 1996; Jalovaara & Miettinen, 2013; Nitsche et al., 2018). Second, studies typically analysed only those at risk of a given birth transition, such as childless couples, when analysing first births (Bueno & García-Román, 2021; Dribe & Stanfors, 2010; Jalovaara & Miettinen, 2013; Nitsche et al., 2018). Hence, these results do not reflect the total effect of education on fertility among partnered and unpartnered individuals. Third, we explicitly adjusted for selection into treatment, a step often omitted in earlier research (e.g., Bueno & García-Román, 2021; Corijn et al., 1996; Jalovaara & Miettinen, 2013; Nitsche et al., 2018). Therefore, the observed associations between couples’ educational pairings and fertility shown in prior studies may not reflect causal effects.

This study also underscores the importance of institutional contexts. In West Germany, where conservative family policies reinforced a gendered division of labour, we found gendered effects of higher education on union formation and fertility: positive for men and negative for women. In East Germany, where housing and employment policies largely decoupled higher education from economic well-being, and family policies supported women’s dual roles as workers and mothers, the effects of higher education on union formation and fertility were smaller and less gendered (Kreyenfeld, 2004; Trappe, 1995).

Our findings have three broader implications for fertility and social stratification research. First, as the negative educational gradient in women’s union formation and marriage has declined or reversed in many high-income countries, the link between education and fertility may also change. If highly educated women increasingly form unions, educational disparities in fertility may narrow over time (Bertrand et al., 2020; Goldstein & Kenney, 2001; Sturm & Van Bavel, 2024). Future research is needed to examine the role of changing union formation patterns in fertility change.

Second, our study contributes to debates about assortative mating and fertility. Although higher education strongly increased the probability of having a highly educated partner, the partners’ education did not mediate the effect of higher education on fertility because the direct effect of partners’ education on fertility was small. If these findings hold in other countries, the profound variation in educational assortative mating across countries might be unrelated to cross-country variation in the educational gradient in fertility (Domański & Przybysz, 2007; Smits, 2003). This underscores the need for further research studying the generalisability of our findings across contexts.

Third, while we focused on cohorts born before the German reunification, studying younger cohorts remains an important task for future research. After reunification, East Germany adopted West Germany’s legal and political systems, but experienced substantial social changes, including rising unemployment, growing inequalities in educational opportunities (Betthäuser, 2019; Burda & Hunt, 2001), and selective out-migration of young women (Leibert, 2016). Meanwhile, West Germany also underwent considerable changes, after reunified Germany introduced policy reforms that expanded public childcare and changed parental leave policies (Geisler & Kreyenfeld, 2019). Together, these developments may have shifted the relationship between education, union formation, and fertility.

This study has several limitations. First, we used a dichotomous measure of education, which captures a key educational boundary but overlooks finer distinctions, such as apprenticeships or shorter and longer tertiary degrees. Nonetheless, this measure suits the analytical framework of this study, which conceptualises education as a treatment. Second, we assessed partner search outcomes based on the education of the most recent partner, which does not capture variation across multiple partners. Although only a small share of individuals were exposed to multiple partners with different levels of education, future research should explore how such partnership histories influence fertility outcomes. Lastly, although we aimed to estimate the causal effect of education on fertility, like most fertility research, we cannot rule out the influence of unobserved confounders. For example, factors such as norms or unmeasured socio-economic conditions could influence educational attainment and fertility behaviour. However, by leveraging available information to control for selection into treatments, our approach strengthens the understanding of how union formation and assortative mating shape the effect of higher education on fertility.

In summary, our findings provide novel insights into the interplay between education, union formation, assortative mating, and fertility. In particular, this study shows that union formation is a key pathway linking higher education and fertility for West German women and men.

## Supplementary Information

Below is the link to the electronic supplementary material.

Supplementary Material 1

## Data Availability

Replication files are available at the Open Science Framework via 10.17605/OSF.IO/2VR4T.

## Footnotes

1 As the literature on this topic is extensive, a comprehensive review exceeds the scope of this section, but excellent overviews were provided by Balbo et al. (2013) and Vasireddy et al. (2023). Here, we focus on key theoretical perspectives that inform our analytical approach.

2 We excluded cases where the partner’s education was missing as well as cases where respondents reported a marriage in the marital history but further information on the partner was not available.

3 Note that we did not include survey weights in the estimation of the IPTWs or the outcome models. The application of survey weights in marginal structural models is an ongoing debate. While some researchers suggest that survey weights are necessary to account for the complex data structure resulting from oversampling or sample attrition (Ridgeway et al., 2015; Robbins et al., 2020), others argue that survey weights are unnecessary and inefficient if the model controls for design variables (Wodtke et al., 2016; Zhao et al., 2021). Since our IPTWs account for migration background and we estimated separate models for women and men from East and West Germany, we control for the main design variables. Therefore, we believe that additionally accounting for survey design weights is not necessary in the context of our study.

4 Stabilised weights should have a mean close to one. Table A2 in Online Appendix demonstrates that this holds in our analysis.
