# Supplementary material for: No Partner, No Children? Union Formation, Assortative Mating, and Educational Inequalities in Fertility in Germany
Source: Eur J Popul. 2026 Feb 3;42(1):8. doi: 10.1007/s10680-026-09766-w (PMC12891275; doi:10.1007/s10680-026-09766-w)
Supplement: Supplementary file 1 — Supplementary Material 1 [file 10680_2026_9766_MOESM1_ESM.docx]

**Online Appendix**

**Appendix A**

Table A1. Propensity score estimation for tertiary education, logistic regression coefficients

| Sample | West German women | West German men | East German women | East German men |
| --- | --- | --- | --- | --- |
| Outcome | Tertiary degree | Tertiary degree | Tertiary degree | Tertiary degree |
| Parental SES |  |  |  |  |
| Parental SES | 0.047^***^ | 0.038^***^ | 0.039^***^ | 0.030^***^ |
|  | (0.004) | (0.004) | (0.005) | (0.006) |
| Missing | 1.523^***^ | 1.391^***^ | 1.680^***^ | 1.028^***^ |
|  | (0.273) | (0.237) | (0.352) | (0.391) |
| Number of siblings  (ref. no siblings) |  |  |  |  |
| 1 sibling | -0.031 | 0.017 | 0.267 | -0.063 |
|  | (0.185) | (0.176) | (0.242) | (0.261) |
| 2 siblings | -0.131 | -0.240 | 0.147 | -0.763^**^ |
|  | (0.194) | (0.187) | (0.266) | (0.307) |
| 3 or more siblings | -0.380^*^ | -0.658^***^ | -0.611^**^ | -0.813^***^ |
|  | (0.205) | (0.189) | (0.291) | (0.295) |
| Missing | -0.394 | -0.660^*^ | -0.206 | -1.345^**^ |
|  | (0.392) | (0.350) | (0.585) | (0.675) |
| Region (ref. large city) |  |  |  |  |
| Medium-sized city | -0.250 | 0.314^*^ | -0.034 | 0.397 |
|  | (0.182) | (0.170) | (0.253) | (0.270) |
| Small town | 0.036 | 0.374^**^ | 0.432^*^ | 0.124 |
|  | (0.162) | (0.159) | (0.241) | (0.265) |
| Rural area | -0.058 | 0.345^**^ | 0.184 | 0.129 |
|  | (0.154) | (0.147) | (0.234) | (0.256) |
| Missing | 0.309 | 0.454^*^ | -0.410 | 0.397 |
|  | (0.282) | (0.268) | (0.554) | (0.467) |
| Migration background (ref. no migration background) |  |  |  |  |
| Direct migration background | 0.439 | -0.019 | -1.394^**^ | 0.262 |
|  | (0.319) | (0.268) | (0.677) | (0.550) |
| Indirect migration background | -0.269 | 0.774^**^ | 0.382 | 0.500 |
|  | (0.368) | (0.384) | (1.475) | (1.016) |
| Constant | -2.839^***^ | -2.099^***^ | -2.479^***^ | -2.030^***^ |
|  | (0.269) | (0.253) | (0.391) | (0.420) |
|  |  |  |  |  |
| Observations | 1,861 | 1,769 | 757 | 710 |

Note: Standard errors in parentheses. Significance: *p < .05, **p < .01, ***p < .001.

Table A2. Summary statistics of weights

|  |  |  | Percentiles | | | |
| --- | --- | --- | --- | --- | --- | --- |
| Weight | Mean | SD | 1st | 25th | 75th | 99th |
| Women, West Germany: |  |  |  |  |  |  |
| All models: |  |  |  |  |  |  |
| *w*(A) | 1.00 | 0.39 | 0.42 | 0.84 | 1.08 | 2.75 |
| *w*(Z) | 1.00 | 0.21 | 0.53 | 0.88 | 1.09 | 1.71 |
| *w*(A) *x w (Z)* | 1.00 | 0.48 | 0.35 | 0.77 | 1.08 | 2.88 |
| C = first child: |  |  |  |  |  |  |
| *W(C)* | 1.00 | 0.08 | 0.87 | 0.97 | 1.01 | 1.28 |
| *w*(A) x *w*(Z) x *W(C)* | 1.00 | 0.47 | 0.32 | 0.75 | 1.08 | 3.07 |
| Men, West Germany: |  |  |  |  |  |  |
| All models: |  |  |  |  |  |  |
| *w*(A) | 1.00 | 0.36 | 0.51 | 0.77 | 1.11 | 2.25 |
| *w*(Z) | 1.00 | 0.16 | 0.62 | 0.91 | 1.07 | 1.49 |
| *w*(A) *x w (Z)* | 1.00 | 0.43 | 0.38 | 0.75 | 1.11 | 2.92 |
| C = first child: |  |  |  |  |  |  |
| *W(C)* | 1.00 | 0.07 | 0.86 | 0.96 | 1.04 | 1.21 |
| *w*(A) x *w*(Z) x *W(C)* | 1.00 | 0.46 | 0.41 | 0.75 | 1.13 | 2.97 |
| Women, East Germany: |  |  |  |  |  |  |
| All models: |  |  |  |  |  |  |
| *w*(A) | 1.00 | 0.36 | 0.48 | 0.77 | 1.10 | 2.33 |
| *w*(Z) | 1.00 | 0.19 | 0.48 | 0.92 | 1.07 | 1.65 |
| *w*(A) *x w (Z)* | 1.00 | 0.47 | 0.38 | 0.75 | 1.11 | 2.47 |
| C = first child: |  |  |  |  |  |  |
| *W(C)* | 1.00 | 0.07 | 0.94 | 0.98 | 1.01 | 1.35 |
| *w*(A) x *w*(Z) x *W(C)* | 1.01 | 0.48 | 0.39 | 0.76 | 1.11 | 2.92 |
| Men, East Germany: |  |  |  |  |  |  |
| All models: |  |  |  |  |  |  |
| *w*(A) | 1.00 | 0.32 | 0.49 | 0.82 | 1.09 | 2.16 |
| *w*(Z) | 1.00 | 0.21 | 0.56 | 0.87 | 1.09 | 1.68 |
| *w*(A) *x w (Z)* | 1.00 | 0.42 | 0.41 | 0.76 | 1.11 | 2.88 |
| C = first child: |  |  |  |  |  |  |
| *W(C)* | 1.00 | 0.06 | 0.84 | 0.97 | 1.02 | 1.15 |
| *w*(A) x *w*(Z) x *W(C)* | 1.00 | 0.40 | 0.43 | 0.76 | 1.11 | 2.69 |

Note: A: tertiary degree, Z: union status (no partner, non-tertiary educated partner, tertiary educated partner), C: first child

Table A3. Total, direct, and indirect effects of having a tertiary degree on being unpartnered, having a highly educated partner, having at least one child, and having at least two children.

|  | Women, West Germany | | | | | |  | Men, West Germany | | | | | | | | |
| --- | --- | --- | --- | --- | --- | --- | --- | --- | --- | --- | --- | --- | --- | --- | --- | --- |
|  | | (1) | (2) | (3) | (4) | (5) |  | (1) | | (2) | | (3) | | (4) | | (5) |
|  | | Z0 | Z2 | C | Y | Realized Y |  | Z0 | | Z2 | | C | | Y | | Realized Y |
| Direct | | 0.040^**^ | 0.281^***^ | -0.078^**^ | -0.014 | -0.066^*^ |  | -0.069^***^ | | 0.244^***^ | | 0.043^*^ | | 0.086^**^ | | 0.099^***^ |
|  | | (0.015) | (0.029) | (0.025) | (0.029) | (0.029) |  | (0.013) | | (0.023) | | (0.020) | | (0.027) | | (0.026) |
|  | |  |  |  |  |  |  |  | |  | |  | |  | |  |
| Indirect | |  |  | -0.023^*^ | -0.010 | -0.025 |  |  | |  | | 0.045^***^ | | 0.038^**^ | | 0.058^***^ |
|  | |  |  | (0.011) | (0.011) | (0.015) |  |  | |  | | (0.012) | | (0.013) | | (0.014) |
|  | |  |  |  |  |  |  |  | |  | |  | |  | |  |
| Via Z0 | |  |  | -0.022^**^ | -0.022^*^ | -0.033^**^ |  |  | |  | | 0.053^***^ | | 0.035^**^ | | 0.061^***^ |
|  | |  |  | (0.008) | (0.009) | (0.012) |  |  | |  | | (0.010) | | (0.011) | | (0.013) |
|  | |  |  |  |  |  |  |  | |  | |  | |  | |  |
| Via Z2 | |  |  | -0.002 | 0.011 | 0.008 |  |  | |  | | -0.008 | | 0.003 | | -0.003 |
|  | |  |  | (0.006) | (0.007) | (0.007) |  |  | |  | | (0.006) | | (0.007) | | (0.006) |
|  | |  |  |  |  |  |  |  | |  | |  | |  | |  |
| Total | |  |  | -0.101 | -0.024 | -0.091^**^ |  |  | |  | | 0.088^***^ | | 0.124 | | 0.157^***^ |
|  | |  |  | (0.053) | (0.076) | (0.030) |  |  | |  | | (0.025) | | (0.128) | | (0.029) |
|  | Women, East Germany | | | | | |  | Men, East Germany | | | | | | | | |
|  | | (1) | (2) | (3) | (4) | (5) |  | (1) | (2) | | (3) | | (4) | | (5) | |
|  | | Z0 | Z2 | C | Y | Realized Y |  | Z0 | Z2 | | C | | Y | | Realized Y | |
| Direct | | -0.028 | 0.239^***^ | -0.023 | -0.017 | -0.031 |  | -0.026 | 0.246^***^ | | -0.022 | | 0.084 | | 0.057 | |
|  | | (0.018) | (0.039) | (0.022) | (0.042) | (0.045) |  | (0.025) | (0.040) | | (0.031) | | (0.048) | | (0.045) | |
|  | |  |  |  |  |  |  |  |  | |  | |  | |  | |
| Indirect | |  |  | 0.024^*^ | 0.032^*^ | 0.045^**^ |  |  |  | | 0.033 | | -0.008 | | 0.013 | |
|  | |  |  | (0.011) | (0.014) | (0.017) |  |  |  | | (0.018) | | (0.014) | | (0.019) | |
|  | |  |  |  |  |  |  |  |  | |  | |  | |  | |
| Via Z0 | |  |  | 0.016 | 0.010 | 0.019 |  |  |  | | 0.017 | | 0.008 | | 0.017 | |
|  | |  |  | (0.010) | (0.007) | (0.012) |  |  |  | | (0.016) | | (0.009) | | (0.016) | |
|  | |  |  |  |  |  |  |  |  | |  | |  | |  | |
| Via Z2 | |  |  | 0.008 | 0.022 | 0.025^*^ |  |  |  | | 0.016^*^ | | -0.016 | | -0.003 | |
|  | |  |  | (0.005) | (0.011) | (0.011) |  |  |  | | (0.008) | | (0.012) | | (0.010) | |
|  | |  |  |  |  |  |  |  |  | |  | |  | |  | |
| Total | |  |  | 0.001 | 0.015 | 0.014 |  |  |  | | 0.011 | | 0.076 | | 0.070 | |
|  | |  |  | (0.089) | (0.145) | (0.048) |  |  |  | | (0.068) | | (0.165) | | (0.047) | |

Note: Z0: probability of having no partner, Z2: probability of having a tertiary educated partner, C: probability of having a first child, Y: probability of progressing to a second child, Realized Y: probability of having a second child. Standard errors in parentheses. Significance: *p < .05, **p < .01, ***p < .001.

**Appendix B**

*Weighting*

This section illustrates how we calculated the weights used in this study. For a detailed description of the weighting procedure, see Lawrence and Breen (2016).

First, we calculated weights ${(w}_{A})$ to account for selection into higher education ($A$), where $X$ refers to our set of observable confounders:

|  | $w_{A}=\frac{pr(A=a)}{pr(A=a\vert X)} .$ | (A1) |
| --- | --- | --- |

We applied these stabilised weights before calculating Equations 1 and 2, where $Z0$(no partner) and $Z2$ (highly educated partner) are the potential outcomes. By applying weights, we aim to achieve statistical independence between the covariates and the treatment.

Unlike unstabilised weights, where the numerator is 1, the numerator of stabilised weights reflects the marginal probability of education taking the value $A$ (e.g., for highly educated individuals, this corresponds to the proportion of highly educated individuals in the respective sample). This approach reduces the variance of the weights, which can lead to a more efficient estimation (Hernán & Robins, 2023; VanderWeele, 2009).

Next, we calculated propensity scores to estimate the probability of receiving the treatment based on the observed covariates. For treated individuals, the denominator in Equation A1 corresponds to these propensity scores, while for untreated individuals, it is given by 1 minus the propensity score (Hernán & Robins, 2023; Lawrence & Breen, 2016).

Second, in Eq. 3, where $C$(first child) is the outcome, in addition to selection into higher education ($A$), we accounted for selection into different types of unions ($Z$). To achieve this, we applied the following weights:

|  | $w_{A}\bullet w_{Z} \mathrm{where} w_{Z} = \frac{pr\left( Z=z \vert A=a \right)}{pr\left( Z=z \vert A=a, X,w_{A} \right)} .$ | (A2) |
| --- | --- | --- |

Lastly, for Eq. 4 where $Y$ (second child) is the outcome, we additionally accounted for selection into having the first child. Therefore, we applied the product of the following weights:

|  | $w_{A}\bullet w_{Z} \bullet w_{C} \mathrm{where} w_{C} = \frac{pr\left( C=C \vert A=a, Z=z \right)}{pr\left( C=c \vert A=a, Z=z, X,w_{A},w_{Z} \right)} .$ | (A3) |
| --- | --- | --- |

The summary statistics of these weights are shown in Table A1. Correctly estimated weights have a mean of approximately 1, which holds for our weights.
